# ‘Safety is about partnership’: Safety through the lens of patients and caregivers

**DOI:** 10.1111/hex.13939

**Published:** 2023-12-21

**Authors:** Kerry Kuluski, Maaike Asselbergs, Ross Baker, Katharina (Kathy) Kovacs Burns, Frances Bruno, Marianne Saragosa, Anne MacLaurin, Virginia Flintoft, Lianne Jeffs

**Affiliations:** ^1^ Institute for Better Health Trillium Health Partners Mississauga Ontario Canada; ^2^ Institute of Health Policy, Management and Evaluation, Dalla Lana School of Public Health University of Toronto Toronto Ontario Canada; ^3^ Patients for Patient Safety Canada Ottawa Ontario Canada; ^4^ Healthcare Excellence Canada Ottawa Ontario Canada; ^5^ School of Public Health, Edmonton Clinic Health Academy University of Alberta Edmonton Alberta Canada; ^6^ Alberta Health Services and Patients for Patient Safety Canada Alberta Canada; ^7^ Sinai Health System Toronto Ontario Canada

**Keywords:** caregiver engagement, patient engagement, qualitative, quality, safety

## Abstract

**Introduction:**

Creating safer care is a high priority across healthcare systems. Despite this, most systems tend to focus on mitigating past harm, not creating proactive solutions. Managers and staff identify safety threats often with little input from patients and their caregivers during their health encounters.

**Methods:**

This is a qualitative descriptive study utilizing focus groups and one‐to‐one interviews with patients and caregivers who were currently using (or had previously used) services in health systems across Canada. Data were analysed via inductive thematic analysis to understand existing and desired strategies to promote safer and better quality care from the perspectives of patients and caregivers.

**Findings:**

In our analysis, we identified three key themes (safety strategies) from patients' and caregivers' perspectives and experiences: Using Tools and Approaches for Engaging Patients and Caregivers in their Care; Having Accountability Processes and Mechanisms for Safe Care; and Enabling Patients and Caregivers Access to Information.

**Conclusions:**

Safety is more than the absence of harm. Our findings outline a number of suggestions from patients and caregivers on how to make care safer, ranging from being valued on teams, participating as members of quality improvement tables, having access to health information, having access to an advocate to help make sense of information and having processes in place for disclosure and closure. Future work can further refine, implement and evaluate these strategies in practice.

**Patient or Public Contributions:**

An advisory group guided the research and was co‐chaired by a patient partner. Members of the advisory group spanned patient and caregiver organizations and health sectors across Canada and included three patient partners and leaders who work closely with patients and caregivers in their day‐to‐day work. In the research itself, we engaged 28 patients and caregivers from across Canada to learn about their safety experiences and learn what safer care looks like from their perspectives.

## 1 INTRODUCTION

Creating safer care is a high priority across health systems.^1^ While there is increasing recognition that safety is more than the absence of harm,^2^, ^3^, ^4^, ^5^, ^6^ there remains a focus in health systems on tackling *specific* harms (e.g., falls in hospitals). The Canadian Institutes for Health Information, a pan‐Canadian organization that tracks and reports healthcare data, defines and measures harm in hospitals as: medication‐associated conditions, healthcare‐associated infections, patient accidents (such as falls) and procedure‐associated conditions (such as harm from surgery).^7^

To understand safety more comprehensively, we need to tap into the wisdom, experience and perspectives of patients and their caregivers (e.g., friends or family who provide care to people with care needs, usually on an unpaid basis), views that are often minimized or missed altogether.^8^, ^9^, ^10^ A growing body of literature indicates that patients and their caregivers can help contribute to safer practices and a safer environment; identifying harmful and potentially harmful events that remain otherwise undetected.^11^, ^12^, ^13^, ^14^ Enhancing safety is not an isolated project or endeavour; it requires a culture of collective responsibility that leverages the insights of patients, their caregivers and providers.^5^ Reflecting, learning and adapting in real time to create safer practices and environments is important given the complex context of healthcare.

### 1.1 Safety frameworks and concepts: More than the absence of harm

A more encompassing lens of safety is offered in the Measurement and Monitoring of Safety Framework (MMSF) developed by Charles Vincent and colleagues in the United Kingdom.^2^, ^3^, ^5^, ^15^ The MMSF consists of five safety‐relevant domains, each with guiding questions: *past harm* (has patient care been safe in the past?); *reliability* (are our current systems and processes reliable?); *sensitivity to operations* (is care safe today?); *anticipation and preparedness* (will care be safe in the future?); and *integration and learning* (are we responding and improving?).

The MMSF Framework aligns with other conceptualizations of safety, referred to as Safety I and Safety II. Safety I applies a risk management lens, emphasizing the importance of learning from errors and understanding why things go wrong. Safety II extends Safety I by focusing on the resiliency of healthcare systems, and the importance of attending to ‘what goes right’ and repeating and celebrating the behaviours and actions that contribute to ‘what goes right’.^6^, ^16^, ^17^ Arguably, the MMSF aligns with both a safety I and safety II approach. For example, the MMSF framework includes a focus on causal factors of errors and getting ahead of problems by examining current practices (safety I) as well as creating proactive systems that are resilient and managed well (safety II).

### 1.2 Importance of engaging patients and caregivers to create safer care

To learn from errors and create proactive and resilient health systems, managers and providers need to engage with patients and caregivers at both the direct care level (e.g., creating opportunities for them to share priorities and goals when care planning) and at the organization and system levels (e.g., through co‐design of service improvements and as members of decision‐making tables).^1^, ^18^ Engagement with patients and caregivers in these ways unearths what is working well and what needs to improve in health systems. Patients and caregivers' lived experience in using health services, including what they observe and what they do to mitigate harm, are important sources of knowledge for healthcare teams, managers and leaders to utilize in their quest to create safer care. Patient and family engagement has been emphasized in health system improvement efforts for several decades but is not always effectively and meaningfully operationalized. For example, in the context of patient safety, a systematic review noted that patient and family engagement has the potential to improve patient safety but there is little implementation guidance for practitioners on how to engage patients and their caregivers most effectively in conversations and activities around safety. The authors concluded that further research is needed to unearth priority areas for action within health systems.^19^ More broadly, the WHO Global Patient Safety Action Plan 2021–2030^1^ calls for a greater focus on patients' and caregivers' engagement by learning about safety through patient experiences and co‐developing solutions with them.

### 1.3 Looking at safety beyond physical harms

The relational aspects of care (feeling heard, appreciated and valued) is at the forefront of patients' and caregivers' expressed needs.^20^, ^21^, ^22^ These relational aspects of care can extend further to a trauma informed approach^23^ which prioritizes physical, emotional and psychological safety; trustworthiness and transparency; peer support; collaboration and mutuality; empowerment of voice and choice; and is attentive to cultural, historical and gender considerations.^24^ The emotional and psychological needs of people were emphasized in a study on hospital deconditioning where harms moved beyond physical deconditioning to mental and emotional harm as expressed by older adults with hip fracture waiting to transfer out of hospital and their caregivers.^25^

In the study reported in this paper, we sought to better understand the safety experiences and perspectives of patients and their caregivers. Based on what they shared, we identified existing and preferred strategies that could potentially improve their safety and the quality of their care. Our overarching question was: ‘What does safe care look like from the perspectives of patients and caregivers?’ A multistakeholder steering committee, co‐chaired by an expert in patient safety and a patient partner, provided oversight and feedback at each stage of the study. The committee had 18 members, three of whom were patient partners. Committee members were from organized patient groups, quality and health councils, health standards organizations, hospital and university settings, and spanned across Canada with two members from the United Kingdom. Some of the committee members had clinical care training and experience; others had personal lived experiences with harm. The committee set the project objectives, helped design (and provided feedback on) the interview guides, supported participant recruitment, provided insight on how to frame the findings and supported this publication.

## 2 METHODS

This qualitative descriptive study utilized individual interviews and focus groups with patients and caregivers to understand what safe care looks like from their perspectives. Before the scheduled interview each participant filled out a demographic survey asking them to state their location (province in Canada), role (patient, caregiver or both), how long they had been in that role, their sex, level of education and degree of financial security. Each interview and focus group started with an overview of the MMSF and a short YouTube clip depicting caregivers' reflections of harm related to a medication error (see Supporting Information for interview and focus group questions). This section of the interview/focus group was facilitated by two members of our research team (A. M. and V. F.). The purpose of this overview was to prepare participants to think about safety with a broader lens (beyond just physical harm). Following that introduction, A. M. and V. F. exited the interview/focus group and the formal interview commenced. The interviews and focus groups were conducted by one doctoral student (F. B.) who is a healthcare professional trained in qualitative research methods. She had no prior relationship with the participants. Two senior researchers (K. K. and L. J.) with expertise in qualitative research methods with many years of experience leading and conducting qualitative research studies mentored her. In the semistructured interview/focus group guide, participants were asked to share their experiences using the healthcare system by drawing on both positive and negative examples. The interviewer asked the following core questions: What has been your experience with using the healthcare system? (probing for both positive and negative examples); When using healthcare, what makes you feel safe or unsafe? What types of things *need to happen* to help you feel safer? See Supporting Information to view the interview/focus group questions.

Our sampling strategy combined purposive and convenience sampling. We drew from two groups of patients and caregivers across geographic regions in Canada: (1) patients/caregivers who were involved in patient partnership activities through formal organizations and networks and (2) patients/caregivers who had recently experienced healthcare from any sector including and not limited to acute, long‐term care, home care, primary care, mental health and addiction care and maternal childcare. We did not conduct targeted recruitment on key characteristics such as sex, gender, ethnicity, age and so on.

Due to the pandemic, all interviews and focus groups were conducted over the telephone or via Zoom. Interviews were audio‐recorded following consent of the participants. The recordings were transcribed verbatim, and the transcripts were checked for accuracy against the audio recordings. Any identifiable information (names, etc.) was removed from the transcripts. Since the goal of the study was to identify what safe care looks like from the perspectives of patients and caregivers and to identify suggestions for safer care, we used an inductive thematic analysis approach to code the interviews and focus groups using a broader definition of safety (as outlined in our background) as a sensitizing concept to guide the selection of relevant excerpts from the interviews and focus groups. A safety strategy was defined as any practice, activity or action that made (or could make) the patient and caregiver feel better about their care experience. Three members of the research team (F. B., K. K. and L. J.) divided up a subset of transcripts to review line‐by‐line and to identify excerpts that identified what participants identified as an existing or desired safety practice. Members of the research team held regular team meetings to discuss findings, based on data coding, and to reach a consensus on a core set of themes and sub‐themes. Data collection and analysis occurred simultaneously until data saturation was achieved (i.e., where no new themes or core ideas were identified in the data).^26^

## 3 FINDINGS

Twenty‐eight individuals participated in the study either through 1‐1 interviews (15 participants) or through focus groups (13 participants spread across four focus groups, each with two to four participants). Table 1 outlines the background characteristics of the participants.

**Table 1:** Participant characteristics.

**Participant type**	**All participants (*n*** = **28)**	**Patients (*n*** = **14)**	**Caregivers (*n*** = **9)**	**Both a patient and a caregiver (*n*** = **5)**
Sex at birth				
Male	29%	36%	33%	100%
Female	71%	64%	67%	
Mean age (range)	56 (21–73) years	52 (21–65) years	56 (27–73) years	54 (25–70) years
Geographic location				
Ontario	64%	57%	67%	80%
British Columbia	14%	21%	11%	0
Alberta	11%	7%	0	20%
Saskatchewan	7%	14%	0	0
Unspecified	4%	0	11%	0
Education level				
Less than high school	4%	0	11%	0
High school	7%	14%	0	0
Trade school/diploma	14%	21%	11%	0
Postsecondary	39%	29%	56%	20%
Graduate degree	32%	21%	22%	80%
Unspecified	4%	7%	0	0
Financial security (difficulty making ends meet)				
Never	61%	50%	67%	80%
Sometimes	21%	36%	11%	0
Usually	7%	0	22%	0
Always	11%	14%	0	20%

Patients and caregivers identified several core issues regarding their safety experiences. This included: feeling silenced or ignored, being excluded from day‐to‐day discussions with the care team (and thus losing the opportunity to flag important details about needs or concerns), providers not circling back or closing the loop following safety incidents and providers not owning up to mistakes or being open to feedback. Patients and caregivers also had difficulty accessing information and adding information to existing health records. Participants noted that their health system lacked reliable and up‐to‐date patient information including patient baseline status, particularly in the emergency room.

Based on these experiences and perceptions as patients and caregivers, the participants identified a number of strategies to improve safety. The strategies were organized into three core themes: (1) using tools and approaches for engaging patients and caregivers in their care; (2) creating and using accountability processes and mechanisms for safe care; and (3) enabling access for patients and caregivers to information and resources. These themes and their subthemes are detailed below with illustrative quotes. Truncated quotes are shown as […].

### 3.1 Theme 1: Using tools and approaches for engaging patients and caregivers in their care

Patients and caregivers wanted to be engaged in meaningful ways with their care teams. This included having regular check‐ins with the team (including when isolated at home), being heard by the team and having their perspectives incorporated into care planning. Having communication guides or tools was also desired by patients and caregivers. These insights and supporting quotes are shared below.

#### 3.1.1 Engaging patients/caregivers in meaningful ways

Several methods were proposed to meaningfully engage patients and caregivers, including having the care team meet with them regularly, to garner their input into system and tool design; including them in bedside reporting; and co‐developing safety care plans. In the quote below, a participant describes their desire for family members to be active members of the care team:

…I think just in the long term care situation, you've got to have regular meetings with family, you've got to have them as part of the care team and not just as sort of here's the care team and the family meets with them. (Participant Interview 14)

#### 3.1.2 Acknowledging what matters to patients/caregivers and incorporating this information into their care

Actively listening and responding to patients and caregivers was seen as validating their concerns, acknowledging them as experts and responding to their treatment preferences. Patients and caregivers wanted to have their needs/concerns followed up on, have regular check‐ins about whether they felt safe or unsafe, and be asked what they needed to feel comfortable.

A participant discussed the importance of welcoming concerns and questions from patients and caregivers as a way to build trust:

…Safety—to me it's about trusting that they got it right. And that trust has to be, it doesn't just happen right? Safety is about them welcoming concerns and questions. And if I need to say ‘Um, excuse me, but what is this drug that you're proposing that I take […]?’ Right? To make sure it's not one of the 200 things what you know. And they need to basically say ‘I hear you. Thank you for asking.’ You know we need to be partners. Safety is about partnership. (Participant Interview 11)

A caregiver discussed a positive care experience with a specialist who acknowledged her as an expert. Honouring her voice made her feel safe.

‘You are the world's leading expert in [your son].’ He [the specialist] said ‘I might be, you know, very knowledgeable in neurology’ … but he said ‘You're the world's leading expert in [your son]. So whatever you have to say I want to hear’ and, you know, let me know that he really respected whatever it was I had to—you know, whatever concerns I had to express or anything like that….

She later went on to say…

When that's happening and it's going well, that makes me feel safe. It gives me some confidence to be able to contact the doctor, go in there for an appointment and say ‘This is what I'm seeing and I'm concerned about it.’ (Participant Interview 3)

She appreciated how the team adapted their practice to create comfort for her son. In this particular case she asked that they sing during unpleasant procedures:

So, we requested that whenever the [care team] came into the room that they hummed, or sang, or did something like that. … one of the questions actually was how to help him be comfortable through unpleasant procedures, and we said sing. You have to sing. They actually put that up on his whiteboard, that it was a note thing, and they sang through every unpleasant procedure… they had the whole team singing when they had to pull the tube out for the ventilator, because that would have been unpleasant. And boy, did that mean a lot to us because not only did they hear [us], [but] they [also] overcame their own personal embarrassment in singing in front of their team members to take care of the patient. (Participant Interview 3)

#### 3.1.3 Using communication strategies and tools to inform and empower patients/caregivers

Patients and caregivers want tangible communication aides to support their care experiences. Examples included: a list of specific questions to ask the care team or a story template for patients to fill out as a way to share ‘what matters to them’. One caregiver also noted the utility of having a ‘cheat sheet’ for the emergency room consisting of key medical baseline information of the patient to avoid any misunderstandings.

—I call it my Emergency Room one‐pager. And it's a one‐page sheet that gives the pertinent basic information, you know, his name, his date of birth, his healthcare number, all that kind of stuff. And then I went on to describe what [son] is normally like; that he's normally very happy and very content. He's normally extremely energetic, talking about what his usual personality is like and what his capabilities are like. (Participant Interview 3)

#### 3.1.4 Checking in on patients/caregivers

Some participants suggested that members of the care team should conduct regular check‐ins with patients, especially those who live alone. These check‐ins could be done by volunteers during long waiting periods, such as in the emergency room. Aside from regular check‐ins, participants recommended following up with patients and caregivers to provide emotional support after safety incidents (including after death and other traumatic events).

A bereaved caregiver who also identified as a patient noted that a simple call just to ask her how she was doing would make her feel safer, since she lived alone and feared having an accident:

A regular call every morning or every afternoon or every evening to check to see if I'm OK. That would make me feel more safe. (Patient Interview 9)

### 3.2 Theme 2: Having accountability processes and mechanisms for safe care

Transparent processes for reporting, disclosing and learning from errors were recommended by participants. It was also important for patients and caregivers to ask questions about recommendations and seek clarification.

#### 3.2.1 Having transparent processes for reporting, disclosing and learning from errors

In cases where patients and caregivers experienced harm, they emphasized that they wanted providers to disclose their errors. Having an opportunity to debrief, seek closure and explore the next steps following an error was important. Several ways of providing feedback were recommended including surveys, interviews, incident reports, root cause analysis, patient feedback (e.g., via patient relations) and public‐facing incident reporting systems. Engaging patients and caregivers in safety event analysis and risk management teams was also recommended. The following quote from a patient reiterates the need for healthcare providers to recognize and disclose errors to patients and caregivers:

I think in order for the patient to feel safe, personally I think I much prefer[the] doctor tells me what went wrong […] You know what happened has happened. But when they speak up it doesn't happen again to others. Of course doctors want to protect themselves, but a lot of times actually when they say it, acknowledge it, the patient would accept. You know because I understand doctors are human, right? They can give the wrong order. (Participant from Focus Group 1)

#### 3.2.2 Patients and caregivers asking questions

Patients and caregivers wanted access to safety reporting systems to share their safety concerns and have their feedback inform system improvements. Speaking up was seen as important, particularly in anticipation of things that could potentially go wrong.

One participant described a desire to work with the care team to come up with joint or shared learning opportunities:

And I've pushed for that, not to sort of rub the noses of the practice in the problems they're experiencing, but to sort of say, hey, you know, you've got patient partners at the table, take all the identifying information away so that, you know, nobody knows who we're talking about, or which provider we're talking about, and let us help you puzzle through, you know, the situation, and ask some questions that may not have occurred to you, and provide support as a council, you know, for changes in how things were done to avoid situations. So in other words you're not working with us as the enemy. (Participant Interview 5)

Another participant noted that both patients and providers have a responsibility to educate one another.

… As a patient, I feel it's up to me to educate my caregivers, my professional caregivers of my condition of my body and what's working, what's not working and the history of it. And I think it's also, in reverse it's up to the caregivers [care providers] to educate me on the treatments that would be necessary for any condition that I might be suffering at the time. And that's why I constantly always say that communication and education can solve many problems and go towards reducing harm. (Participant Interview 13)

A participant who was a caregiver for her teenage son shared the following about how she coached him to speak up to staff, which could mitigate risk:

‘Make sure, when they give you that urine cup that your name and label is on.’” He had a hyphenated name […]I said ‘Make sure it matches’ and I trained him [to do] that. So, what do you know, at one [hospital] he had a scan and I was at work. He told me, ‘Mom, I had to go to new care medicine to get—’ I forget which scan this was now, some kind of scan, ‘But it looked different on the requisition.’ So, he was not shy enough, so, when he got to the new care medicine department in imaging, he actually said ‘I was told by my doctor I'm having this scan, but this piece of paper says another scan.’ Then, the MRT says ‘You're actually right. That is not the same scan.’ (Participant Interview 1)

This participant reiterated the importance of having a safe space to speak up:

Patients have a lot of questions typically about medications, but a lot of times they don't feel empowered or—what's the word I'm looking for—I'm going to use a word that's probably on the radar—they don't feel it's safe to ask their questions, and for fear that the doctor won't care, or will think it's a stupid question, or will minimise their concerns, or be very dismissive in terms of what they say. Now, some of that could be on the patient in the sense that, you know, if you don't ask, how's the doctor supposed to know? They're not mind readers. So there's a balance here for what patients to be responsible, active partners in their care need to do, but there's also the practice on the other side where their physicians, you know, create conditions where people are likely to be willing to share their experience. (Participant Interview 5)

### 3.3 Theme 3: Enabling patients and caregivers access to information

The final theme was about providing patients and caregivers with access to their personal health information. This also entailed having ‘an advocate’ (i.e., point person) for patients and caregivers to connect with, such as a healthcare provider or representative who could be easily contacted; enabling access to and ownership of health information; and providing educational sessions on safety.

#### 3.3.1 Having an advocate or point person

To support patients and caregivers in navigating their care and accessing information it was recommended that an advocate (or point person) be available. This person could be their most responsible provider or another designate (clinician, liaison or volunteer). This is particularly important during care transitions or for assisting socially isolated patients and caregivers. Relatedly, advocates (someone to help patients learn what to ask and how) were recommended by a participant who was a caregiver for her husband.

I think advocates, asking questions that you might not think of, pressing for information they might not be comfortable with. (Participant Interview 11)

#### 3.3.2 Enabling access to and ownership of information

Participants expressed their desire to have access to the patient's medical records in real‐time. A participant who was caring for her mother described the practicality of having all of their medical histories of any patient readily available during their care interactions in different settings:

… But that's another thing, like all that stuff should be available, right? If I show up at the ER [Emergency Room], I would like to think that my OHIP [health insurance] card would give access to the ER doctor and my history and everything, not having to count on having to call another doctor or get a fax, right? Or at least listen to me, like take the time to do the history. (Participant from Focus Group 3)

Another participant who identified as both a patient and caregiver noted:

If there was a website you could go in and as a caregiver or as a signed‐over caregiver put in some kind of code and be able to see the full reports online, just to be able to have it there for your reference, that would be probably the coolest addition to a healthcare system. And I think that would be very progressive and it could help to address a lot of the old, old systematic problems that we have. (Participant from Focus Group 4)

#### 3.3.3 Providing educational sessions on safety

Educating patients and caregivers on safety was deemed important and could take on many forms including educating patients and caregivers on the MMSF to get a broader understanding of safety; providing educational leaflets, videos and sessions to patients and caregivers on how they can be involved in patient safety; providing patient awareness videos on stories of safety and harm; holding awareness campaigns; providing training for advocates (e.g., point person); and, for treatments, having a provider such as a pharmacist discuss side effects to educate patients and caregivers on potential adverse reactions and drug–drug interactions.

Providing safety education and training to advocates (not just patients and caregivers) was recommended by a participant:

… Well, certainly if we were to speak of third party advocates, that they would get sufficient training and the education so that they could bridge the gap between the professionals and the patient. And you know that's a training cycle that I don't know if anybody has really developed. (Participant Interview 130

## 4 DISCUSSION

The dominant perspective and definition of patient safety is ‘the absence of harm’. Measures of safety are based on the number of adverse events, and safety strategies derive from the analysis of those events and the identification of ways to reduce system and individual vulnerabilities that permit harm. While this approach to safety continues to be valuable, it is designed to improve safety and learning *after events occur*. This paper outlines what safe care presently looks and feels like from the perspectives of patients and their caregivers, and includes their ideas and aspirations for safer healthcare.

Many of our findings align with the MMSF.^2^, ^3^ For example, patients and caregivers recognized when circumstances did not feel right, and caregivers anticipated when a situation could go wrong, based on their knowledge and history of the patient. This is an example of *anticipation and preparedness* (an MMSF domain). By incorporating the viewpoints of patients and caregivers, their specific needs (which might not be understood or anticipated by the care team) can be incorporated into the care plan or acted on in real time.

When patients and caregivers are not part of ongoing discussions with care teams there can be missed opportunities to operationalize *all* MMSF domains: *past harm* (unpacking what has gone wrong); *reliability* (assessing whether current systems and processes are consistent and working well); *sensitivity to operations* (addressing safety in real time); *anticipation and preparedness* (determining how care can be safe in the future); and how knowledge of all of these elements can be integrated to support a learning culture (*integration and learning*).^27^

Patients and caregivers wanted more opportunities to engage with their care teams in regular discussions about their care. In previous research, bedside rounding and huddles provided opportunities for patients and caregivers to interact with care providers, provide input and become more informed about their care.^28^ Involving patients and caregivers in care team discussions allows all parties to stay informed and offer different perspectives. Patient involvement in reporting safety issues offers perspectives that tend to differ from the incidents reported by providers, providing a fuller picture of contributors to unsafe care. Hernan et al.^29^ examined such differences in patient‐ versus provider‐reported incidents in their patient safety study in primary care settings in England and Australia. The authors noted that previous research (using nonpatient reported sources) pointed to communication, diagnoses and medication errors as common safety issues in primary care whereas access to care (particularly timely access to physicians) was the most commonly reported safety issue identified by primary care patients.

Healthcare providers and organizations need to promote psychological safety for patients and caregivers so they can speak up without fear of reprisal. Environments that support psychological safety for care providers enable them to be more open and transparent (admitting to mistakes and working on solutions). For example, Murray et al.^30^ noted that when providers feel psychologically safe, they are more comfortable raising concerns, acknowledging mistakes and expressing uncertainties. Greater transparency overcomes perceived resistance from care providers to disclose an error. Standardization in reporting and disclosing errors and a ‘learning‐from’ approach (instead of punitive approaches) to errors also promotes safety. Overall, patients and caregivers guided us to understand that safety is not just about a series of activities and checkboxes. Safety is an emotional experience and attributes like vulnerability and empathy should be core competencies among care teams.

In addition to attending to the emotional aspects of care, there are specific healthcare *tools*, most notably, information systems and health records that could ideally be accessed in real time to support patients and caregivers as members of the care team (and could address our participants' concerns about poor access to information). For example, tools such as MyChart and OpenNotes are integral parts of some Health Information Systems including EPIC (which has been implemented in health organizations across North America). Tools like MyChart and OpenNotes are viewed favourably by patients, allow them to access their records,^31^ promotes transparency and improves trust with their providers^32^ with the benefits outweighing perceived risks.^33^ Importantly, OpenNotes have provided an important mechanism for patients to report medical errors and inaccuracies directly into clinical notes.^34^ In a study conducted by Bell et al.,^35^ over 6000 patients were invited to provide feedback on clinical notes for a quality improvement study. Patients and caregivers reported safety concerns and potential errors in approximately one‐quarter of reports. Over 60% of these cases were confirmed to be actual errors or safety threats by providers and patients confirmed that over half of these reported errors led to changes in care. The Danish Patient Safety Database is an example of a broader reporting system where patients can voluntarily provide safety incident reports from their perspectives.^36^ Comparison of patients' reports to those of care providers reveals that patients provide more detail, emphasize the relational aspects of care and provide needed context to fully unpack the ‘chain reactions’ of issues across time and systems of care.^36^

In addition to having mechanisms and tools to contribute or obtain information, we also learned that information provided to patients and caregivers needs to be digestible, meaningful and easy to navigate. Access to an advocate who could help patients with sense‐making and interpretation of data would also promote safety. From an organizational perspective, the feasibility of adding a new role (i.e., advocate) or additional tasks to a strained workforce is likely difficult. Alternatively, examining and buffering current resources in the system (care providers who may already take on these tasks formally or informally) and/or the volunteer sector (including retired healthcare professionals) and training them to elicit ongoing discussions and liaise between providers and patients may be an option worthy of consideration. Those patients who do not have access to a caregiver (e.g., an involved family member or friend) should be prioritized for this extra support. Importantly, patients, caregivers and advocates can support teams with *integration and learning* by providing their lens on patient safety issues and experiences, so others do not suffer the same harms they experienced. Leveraging the roles of patient advocates and safety champions is one of the core recommendations in the Global Patient Safety Action Plan 2021–2030.^1^ Without these approaches, tools and activities, we run the risk of providers, patients and caregivers resorting to *work‐arounds*—such as creating their own tools (patient paper summaries) to have on hand in case of emergencies, or the reliance on caregivers and advocates to fight for what is needed and settle injustices on an ad hoc basis.

Patient partnership strategies can also promote safety.^18^ Some of the participants in our study were involved in such patient partnership activities or desired greater involvement in decision‐making and quality improvement at the organizational level to contribute to root cause analysis discussions and planning for better systems of care. Patient partnership in quality improvement activities was explored in a scoping review by Cox et al.,^37^ who provided insight into the key capabilities that organizations require to fully embrace patient partners in these roles. These key capability domains included: personal attributes (e.g., being dedicated to improving healthcare); relationships and communication (e.g., recognizing the value that each team member brings and advocating for each other); philosophies, models and practices (e.g., implementing best practices in patient engagement by providing ongoing support and feedback to patient partners). Sharing power was an overarching domain that entailed supporting patient‐led approaches, transforming power dynamics and encouraging shared decision‐making. This type of framework points to key ingredients that could enable more fulsome patient engagement and input, which is key to actioning the MMSF domains and creating spaces to talk about and address safety concerns.

Healthcare organizations need to cultivate and reward a culture that normalizes the reporting of negative experiences. While workarounds can be helpful in some circumstances, they also reduce *reliability*.^38^ More broadly, healthcare organizations that adopt the principles of Highly Reliable Organizations (HROs) continually anticipate risk and pay attention to the “small things,” which could cascade to major problems. HROs continually examine the root causes of problems and attend to the insights of front‐line personnel.^39^ Merchant et al.^39^ highlighted a need to honour *all* levels of expertise regardless of position and rank within an organization. A highlighted example is a patient who received the ‘Good Catch’ award for raising several safety issues on the mental health ward where he was receiving care, an example of honouring patient expertise and their commitment to safety, as opposed to only capturing the care provider perspective.^39^

Our findings outline a number of suggestions from patients and caregivers on how to make care safer, ranging from being valued on teams, participating as members of quality improvement tables, having access to health information, having access to an advocate to help make sense of information, having processes in place for disclosure and closure and so on. Working with consistent providers who demonstrate compassion and treat patient and caregivers experience and accounts as legitimate input are core ingredients for a safer care environment. However, none of these initiatives will hold much weight if not embedded within enabling organizations as described above. Some of the suggestions in our study are quite broad (e.g., helping patients and caregivers feel valued on teams) and could be further refined into actionable strategies. In future research, such refinements, including delineating short‐term actions, based on priorities and resource capacity of local health organizations, could be a helpful approach to initiate improvement. These strategies could be implemented, tested, evaluated and refined in future research.

## 5 LIMITATIONS

Our sampling approach included recruiting from patient and caregiver organizations, which may have introduced bias in our sample in favour of individuals with more experience partnering in healthcare activities; however, this breadth of experience also helped us unearth safety strategies across the spectrum of engagement.^18^ We did not have participants from every province or the territories or consider how geography (such as living in isolated areas of the north) affected people's experiences of safety. We did not collect data on ethnicity or use an equity, and diversity framework or criteria in our sampling. In other words, our recruitment approach did not consider a more balanced selection of participants across geography, age, sex, gender, education, racialization and ethnicity. Our findings are thus limited in their transferability and future work will be needed to better understand safety for the communities and people not represented in this work. Also, we inductively coded the data and briefly reflected on how our findings related to the MMSF domains in the discussion. Future research could include the MMSF as an analytic framework to gain a more comprehensive picture of how patient and caregiver safety experiences and suggestions align with the framework, including gaps and areas for refinement.

## 6 CONCLUSIONS

Safety is more than the absence of harm. In this paper, we share accounts from patients and caregivers on their healthcare experiences honing in on what safe care looks like from their perspectives.

We presented the experiences and insights from patients and caregivers as strategies that could potentially enable safer care. These strategies provide rich examples of proactive, collaborative ways in which safety can be a core part of people's experience, in which they are involved, in real time and not just reflecting on harm after the event occurs. The insights shared by patients and caregivers offer tangible strategies that could be further refined and implemented in health systems and evaluated in future research. There is also an opportunity to dive deeper into specific care settings (e.g., primary care, home care, long‐term care, etc.) to understand how patients and caregivers perceive safety.

## AUTHOR CONTRIBUTIONS

Kerry Kuluski: co‐led the study with Jeffs, supported data analysis, led the drafting and re‐drafting of the manuscript and submission. Maaike Asselbergs: advisory role, supported recruitment, data interpretation and editing of the manuscript. Ross Baker: advisory role supported data interpretation and editing of the manuscript. Katharina (Kathy) Kovacs Burns: advisory role, supported recruitment, data interpretation and editing of the manuscript. Frances Bruno: led recruitment, data collection and supported the analysis and editing of the manuscript. Marianne Saragosa: supported the preparatory phases of the study and editing of the manuscript. Anne MacLaurin: advisory role, supported recruitment, data collection, data interpretation and editing of the manuscript. Virginia Flintoft: advisory role, and supported data collection, data interpretation and editing of the manuscript. Lianne Jeffs: co‐led the study with Kuluski, and supported data collection, analysis and manuscript revisions.

## CONFLICT OF INTEREST STATEMENT

One of the co‐authors (MacLaurin) is employed by the study funder. While she was involved in introducing the framework to study participants, she did not conduct the interviews or focus groups nor play a lead role in data analysis.

## ETHICS STATEMENT

Ethics approval was received by the University of Toronto Ethics Review Board (#38877) and the Sinai Health System Ethics Review Board (#20‐0047‐E). All patient and caregiver participants consented to participate in the research.

## Supporting information

Supporting information.

## Data Availability

Data are not available for use outside the research team, who produced the study reported in this paper, since such use was not included in the Research Ethics Board (REB) applications and when consenting participants.
