# Supplementary material for: ‘Safety is about partnership’: Safety through the lens of patients and caregivers
Source: Health Expect. 2023 Dec 21;27(1):e13939. doi: 10.1111/hex.13939 (PMC10739088; doi:10.1111/hex.13939)
Supplement: Supplementary file 1 — Supporting information. [file HEX-27-e13939-s001.docx]

**Supplemental File:** *Focus Group/Interview Questions*

**Fervid’s Story Video shared in advance of the interview / focus group**

<https://www.patientsafetyinstitute.ca/en/toolsResources/Member-Videos-and-Stories/Pages/Fervids-legacy-of-care-lives-on-through-loved-ones.aspx>

**General Questions**

1. **What has been your experience with using the health care system?**
   1. We would like to hear examples of both positive and negative experiences. You can reflect on any of your previous encounters with the health care system. This can be experience of receiving care for yourself, or you supporting a family member (child, parent, friend) through their health care encounter.

*Prompts:*

- - 1. Thinking back to a previous experience using health care, do positive experiences come to mind? Reflect on one specific experience and let us know what was happening to make it positive? What worked well and how did it make you feel?
    2. Thinking back to a previous experience of health care, do negative experiences come to mind? Reflect on one specific experience and let us know what was happening to make it negative? What went wrong and how did it make you feel? How could it have been better?

1. **Diving into their experiences and feelings about safety:**
   1. **When using health care, what makes you feel safe or unsafe?**
   2. **What types of things *need to happen* to help you feel safer?**

*Prompt:*

What can *you* do or could you have done to help contribute to your safety (or your loved one’s) when using the health care system? (Pull from their own story / experiences described)

1. **Have you or a loved one been harmed while receiving health care. Refer to the slide- expanded view of harm and see what examples they can come up with.**

*Prompts:*

- 1. Tell us about that experience
  2. Was there any follow-up with you about how/and if the issue was resolved?
  3. What key insights/learnings did you take from this experience?
  4. From your understanding, what key learnings/insights did your clinicians/health care organization take away?
  5. What can be done differently next time?

1. **Now when you hear the word safety, what comes to mind?**
2. **Thank you for sharing your experiences today. Is there anything more that you would like to add before we close the session?**
